# Effect of Dentin Wetness on the Bond Strength of Universal Adhesives

**DOI:** 10.3390/ma10111224

**Published:** 2017-10-25

**Authors:** An-Na Choi, Ji-Hye Lee, Sung-Ae Son, Kyoung-Hwa Jung, Yong Hoon Kwon, Jeong-Kil Park

**Affiliations:** 1 Department of Conservative Dentistry, School of Dentistry, Pusan National University, Dental Research Institute, Yangsan 50612, Korea; casual86@hanmail.net (A.-N.C.); song-ae@hanmail.net (S.-A.S.); 82dreamy@hanmail.net (K.-H.J.); 2 Department of Oral Pathology & BK21 PLUS Project, School of Dentistry, Institute of Translational Dental Sciences, Pusan National University, Yangsan 50612, Korea; jihyelee@pusan.ac.kr; 3 Department of Dental Materials, School of Dentistry, Pusan National University, Yangsan 50612, Korea; y0k0916@pusan.ac.kr

**Keywords:** bonding, adhesive resins, dentin, micro-tensile strength, confocal laser scanning microscopy

## Abstract

The effects of dentin wetness on the bond strength and adhesive interface morphology of universal adhesives have been investigated using micro-tensile bond strength (μTBS) testing and confocal laser scanning microscopy (CLSM). Seventy-two human third molars were wet ground to expose flat dentin surfaces. They were divided into three groups according to the air-drying time of the dentin surfaces: 0 (without air drying), 5, and 10 s. The dentin surfaces were then treated with three universal adhesives: G-Premio Bond, Single Bond Universal, and All-Bond Universal in self-etch or etch-and-rinse mode. After composite build up, a μTBS test was performed. One additional tooth was prepared for each group by staining the adhesives with 0.01 wt % of Rhodamine B fluorescent dye for CLSM analysis. The data were analyzed statistically using ANOVA and Tukey’s *post hoc* tests (α = 0.05). Two-way ANOVA showed significant differences among the adhesive systems and dentin moisture conditions. An interaction effect was also observed (*p* < 0.05). One-way ANOVA showed that All-Bond Universal was the only material influenced by the wetness of the dentin surfaces. Wetness of the dentin surface is a factor influencing the micro-tensile bond strength of universal adhesives.

## 1. Introduction

Dental adhesive systems can be classified into two main categories according to their adhesion strategy: “etch-and-rinse” and “self-etch” systems [1,2].

Etch-and-rinse adhesives, known as total-etch adhesives, usually use 35% to 37% phosphoric acid to etch enamel and dentin, followed by thorough water rinsing of the etched surface [1]. On enamel, the acid removes the smear layer from the enamel surface and demineralizes the superficial hydroxyapatite to reveal the etched enamel prisms [1]. The adhesive then penetrates the etched enamel prisms to form resin microtags that interlock with superficial enamel porosities created by the etching procedure [1]. On dentin, the acid demineralizes the superficial hydroxyapatite and removes the smear layer and smear plugs to expose the collagen fibrils that remain anchored into the underlying mineralized dentin and open the dentinal tubules, funneling their orifices [1]. Although the etch-and-rinse strategy is believed to be reliable and predictable for bonding to enamel, there are some difficulties in bonding to acid-etched dentin because of its morphological, histological, and compositional complexities [1]. The mechanism of bonding to dentin is primarily diffusion-based and depends on the hybridization or infiltration of adhesive resin within the exposed collagen mesh [3]. The exposed collagen fibrils collapse after air-drying the dentin surfaces [4], which might interfere with adhesive resin infiltration and lead to a decreased bond strength [5]. Therefore, surface wetness is believed to be important for demineralized dentin to avoid collapse of the collagen fibrils [4]. Although the integrity of the collagen network can be recovered partially by rewetting the substrate [5,6] with acetone-based, ethanol-based, and/or water-based adhesives [7,8], controlling the moisture on the dentin surface and maintaining the original structure of collagen fibrils are highly technique-sensitive [9].

Self-etch adhesives contain acidic monomers that can dissolve the smear layers, and act simultaneously as a conditioner and primer on the enamel and dentin without the need for a separate phosphoric acid etching step [10,11]. These adhesives render a smear layer permeable to monomers rather than removing it completely [12,13]. According to their acidity, these adhesives can be classified as strong (pH < 1), intermediately strong (pH = 1 to 2), mild (pH ≈ 2), and ultra-mild (pH > 2.5) [14]. The acidity and chemical composition of the adhesives are strongly related to their interaction depth in dentin and, accordingly, the morphological features of the adhesive interfaces [12,13,14]. Self-etch adhesives necessarily contain water to enable the ionization of acidic monomers, whereas solvents, such as ethanol or acetone, are added to accelerate water elimination [15]. Because self-etch adhesive systems do not require moisture before applying the adhesives on the dentin surfaces, they are also called etch-and-dry adhesives [13]. Although these systems have been described as less technique-sensitive with regard to moisture control of the dentin surfaces than etch-and-rinse systems [12,13], the degree of drying dentin before adhesive bonding still has operator-dependent variables.

Recently, a new version of one-bottle adhesive systems called universal or multi-mode adhesives were introduced on the market. These adhesives are considered to be the last generation of adhesive systems, and can be used as an etch-and-rinse, self-etch, or selective-etch strategy [16]. They have some similarities with one-step self-etch adhesives based on the “all-in-one” concept, but they incorporate the versatility of adapting them to clinical situations by application under different etching modes. Some of these adhesives, which contain 10-methacryloyloxydecyl dihydrogen phosphate (10-MDP) or other organophosphate monomers, have the ability to bond chemically to indirect substrates, such as zirconia, glass ceramic, metal, and composite resin without the use of an additional primer [17]. Because these adhesives recently entered the market [17], relatively little information is available on their dentin bonding performance. In particular, the effects of dentin wetness on the bond strength of these adhesives have not been investigated so far.

Therefore, this study examined the effects of the wetness of the dentin surface on the immediate micro-tensile bond strength (μTBS) of resin/dentin bonds mediated by universal adhesives used in the etch-and-rinse and self-etch modes to simulate a clinical application method. The null hypothesis tested was that the wetness of the dentin surface would not influence the immediate μTBS of the universal adhesives.

## 2. Results

### 2.1. μTBS

**Figure 1:**
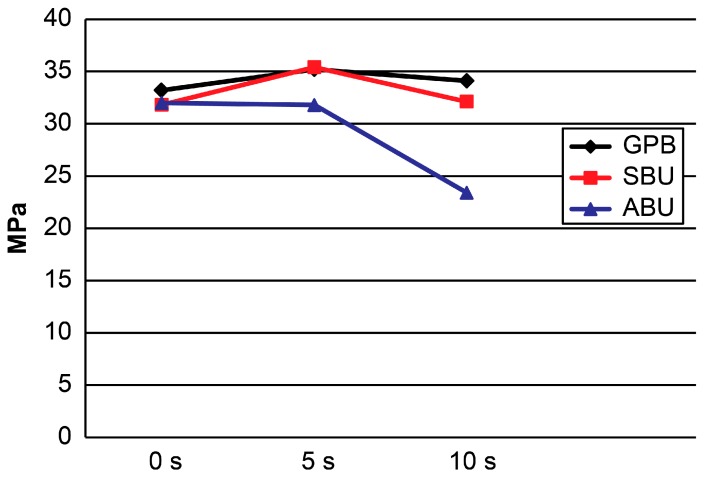
Micro-tensile bond strength (μTBS) means obtained in etch-and-rinse mode. GPB: G-Premio Bond; SBU: Single Bond Universal; ABU: All Bond Universal.

**Figure 2:**
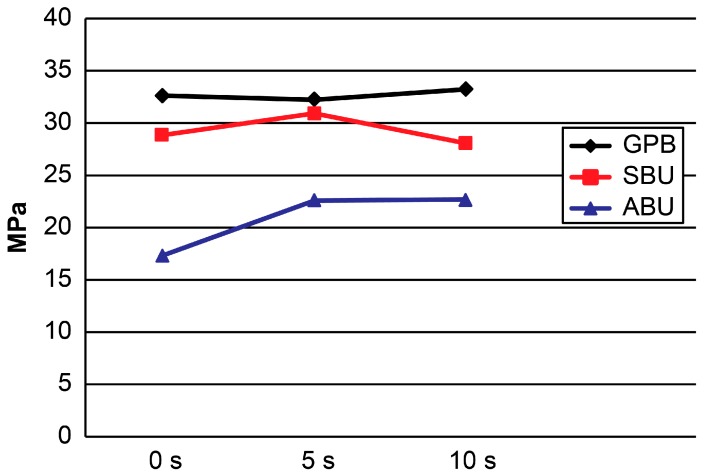
μTBS means obtained in self-etch mode. GPB: G-Premio Bond; SBU: Single Bond Universal; ABU: All Bond Universal.

Table 1 and Figure 1 and Figure 2 present the results. For etch-and-rinse mode, the two-way ANOVA revealed significant differences among the adhesive systems (*p* < 0.001) and dentin moisture conditions (*p* < 0.001). The interaction between these factors was also significant (*p* < 0.001). For self-etch mode, the two-way ANOVA showed the significant effects of “adhesive system” (*p* < 0.001), “dentin moisture condition” (*p* < 0.025), and the interaction between these factors (*p* < 0.018).

Table 1 presents the results from the multiple comparisons obtained by the one-way ANOVA as superscripted letters. In etch-and-rinse mode, there was no significant difference among the different experimental conditions for both SBU and GPB (*p* > 0.05). For ABU, however, the 10 s groups showed a significantly lower mean μTBS than the 0 s and 5 s groups (*p* < 0.05).

In self-etch mode, no significant difference was observed among the different experimental conditions for both SBU and GPB (*p* > 0.05). For ABU, a higher mean μTBS was obtained with the 5 s and 10 s groups and a lower mean μTBS was obtained with the 0 s groups (*p* < 0.05).

### 2.2. Failure Mode Analysis

**Figure 3:**
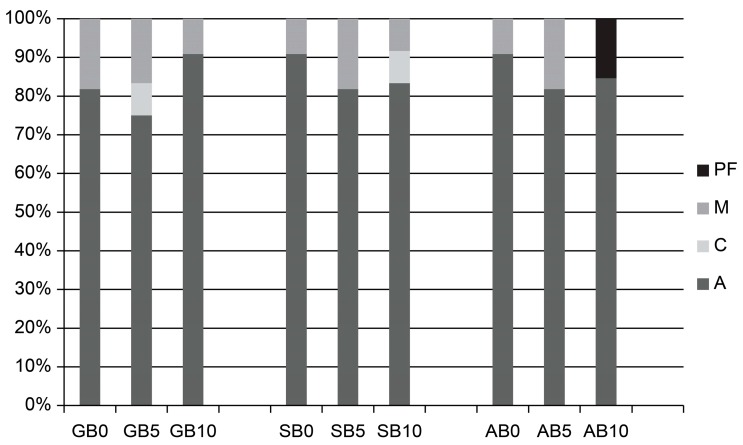
Distribution of the failure modes (%) in etch-and rinse mode. **GB0**: 0 s group for G-Premio Bond; **GB5**: 5 s group for G-Premio Bond; **GB10**: 10 s group for G-Premio Bond; **SB0**: 0 s group for Single Bond Universal; **SB5**: 5 s group for Single Bond Universal; **SB10**: 10 s group for Single Bond Universal; **AB0**: 0 s group for All Bond Universal; **AB5**: 5 s group for All Bond Universal; **AB10**: 10 s group for All Bond Universal. **PF**: premature failure; **M**: mixed failure; **C**: cohesive failure; **A**: adhesive failure.

**Figure 4:**
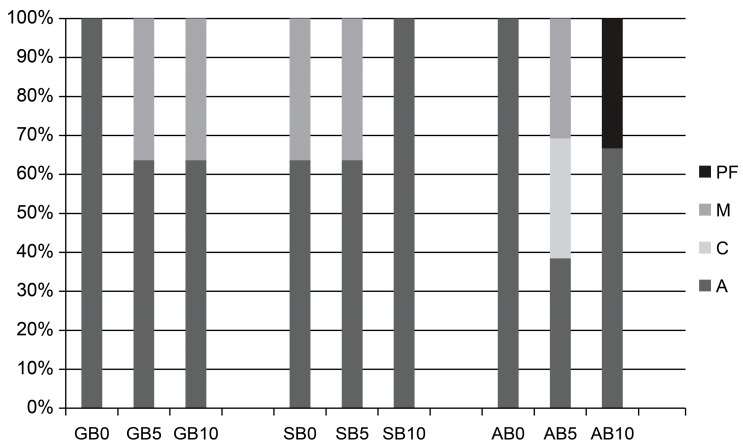
Distribution of the failure mode (%) in self-etch mode. **GB0**: 0 s group for G-Premio Bond; **GB5**: 5 s group for G-Premio Bond; **GB10**: 10 s group for G-Premio Bond; **SB0**: 0 s group for Single Bond Universal; **SB5**: 5 s group for Single Bond Universal; **SB10**: 10 s group for Single Bond Universal; **AB0**: 0 s group for All Bond Universal; **AB5**: 5 s group for All Bond Universal; **AB10**: 10 s group for All Bond Universal. **PF**: premature failure; **M**: mixed failure; **C**: cohesive failure; **A**: adhesive failure.

Figure 3 and Figure 4 present the results of fracture mode analysis. Adhesive failure was observed in all groups, but a more mixed failure was noted in the etch-and-rinse than the self-etch mode.

### 2.3. Confocal Laser Scanning Microscopy (CLSM) Analysis

**Figure 5:**
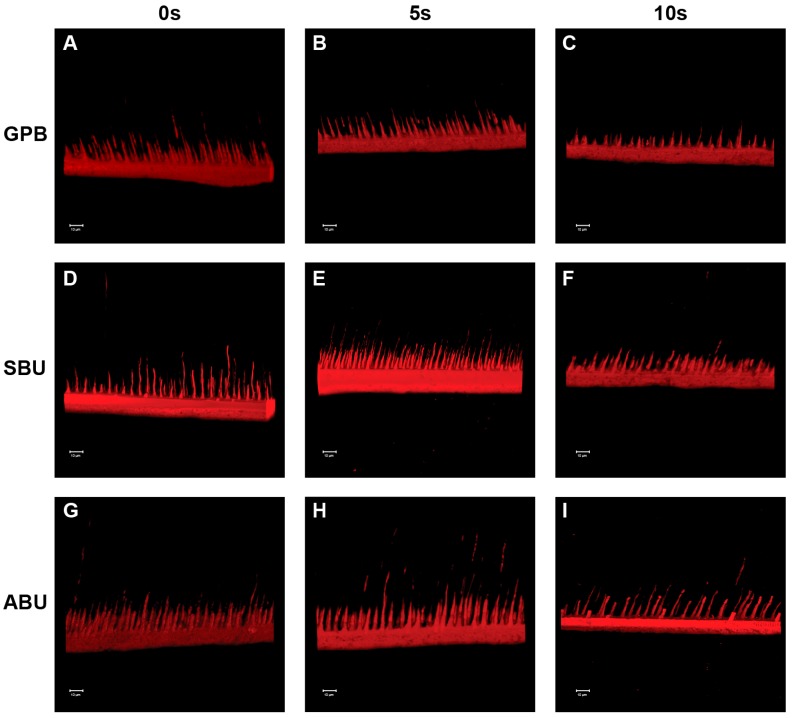
Confocal laser scanning microscopy (CLSM) images of the universal adhesives interfaces in etch-and-rinse mode. (**A**–**C**) correspond respectively to GB0, GB5, and GB10; (**D**–**F**) correspond respectively to SB0, SB5, and SB10; (**G**–**I**) correspond respectively to AB0, AB5, and AB10.

**Figure 6:**
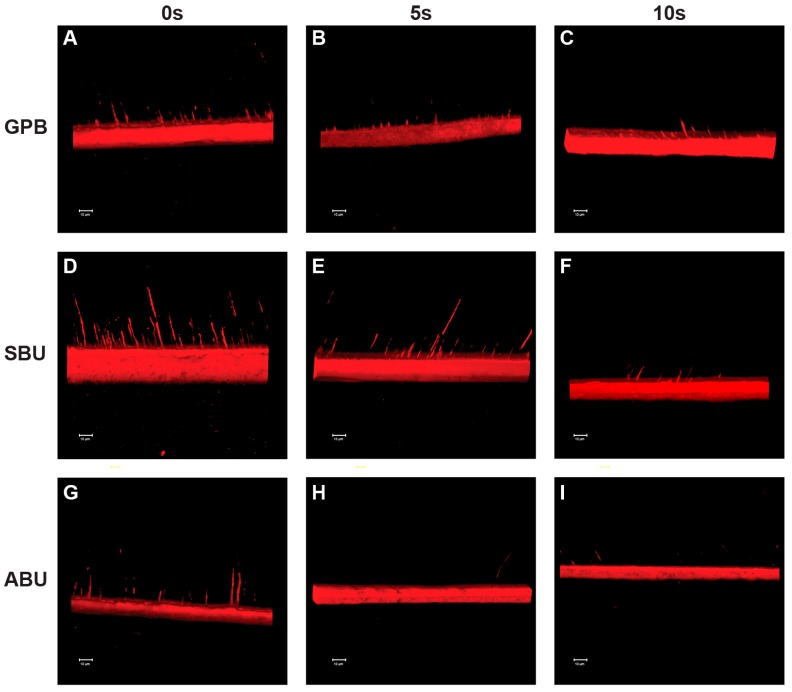
CLSM images of the universal adhesives interfaces in self-etch mode. (**A**–**C**) correspond to GB0, GB5, and GB10, respectively; (**D**–**F**) correspond to SB0, SB5, and SB10, respectively; (**G**–**I**) correspond to AB0, AB5, and AB10, respectively.

Figure 5 and Figure 6 show representative CLSM images of the adhesive interfaces from each experimental group. When the universal adhesives were bonded to phosphoric-acid etched dentin, a hybrid layer was clearly identified and a consistently high concentration of resin tags was formed in the dentinal tubules (Figure 5). On the other hand, prolonged air-drying of the dentin surfaces resulted in an irregular pattern and a low concentration of resin tags as well as a shallow penetration depth (Figure 5 and Figure 6). Although the adhesive thickness was not uniform throughout the adhesive interfaces, this phenomenon was observed more clearly in ABU than GPB and SBU (Figure 5).

When the universal adhesives were bonded using a self-etch protocol, a low concentration of resin tags and thin hybrid layer were observed at the adhesive interfaces compared with the etch-and-rinse mode (Figure 6). No significant difference in the morphology of the adhesive interfaces was observed among the three universal adhesives used in self-etch mode (Figure 6). Regardless of the adhesive systems, the morphology of the adhesive interfaces was similar in the experimental groups (Figure 6).

## 3. Discussion

The rationale behind this study was that when operators are faced with dentin surfaces that contain smear layers and excessive water after cutting or grinding, they attempt to control the degree of moisture of the dentin surfaces, basically depending on their own interpretation of what appears to be the most appropriate moisture condition of the dentin surfaces before the application of universal adhesives in either a self-etch or etch-and-rinse strategy. As these adhesive systems have been introduced recently, however, there are insufficient independent scientific data on their laboratory and clinical performance regarding the moisture control of the dentin prior to the bonding procedures. In addition, the manufacturer’s instructions of commercial universal adhesive systems contain little information on the moisture condition of the dentin surfaces. Therefore, this study examined the effect of the dentin wetness on the bonding property of three universal adhesives. As the bond strength was obtained after 24 h of water storage, it should be considered to be immediate bond strength.

While a two-way ANOVA—using adhesive systems and moisture condition as independent variables—is an appropriate statistical design for the present study, a one-way ANOVA was also applied because the interaction effect could play a role in detecting the influence of wetness on a specific adhesive system. Indeed, one-way ANOVA showed where the differences occurred among the experimental groups. One-way ANOVA showed that ABU was the only adhesive that was actually influenced by the dentin wetness. Therefore, the null hypothesis that the wetness of dentin would not be associated with the immediate μTBS of the three universal adhesives was rejected.

Mineralized dentin is, by volume, composed of approximately 50% inorganic apatite crystals, 30% collagen, and 20% water. In the etch-and-rinse strategy, the surface and subsurface mineral is solubilized, extracted, and is replaced by rinse-water which, combined with intrinsic water, surrounds the collagen fibrils [1]. When the demineralized dentin is air-dried, the collagen fibrils collapse, resulting in reduced adhesive infiltration [6,18]. To overcome this, this collapsed collagen requires water to lower the modulus of elasticity to re-expand and enable the diffusion of monomers into the collagen mesh [6,19]. Therefore, traditional water-wet-bonding technique is thought to be effective to improve the initial bond strength of etch-and-rinse adhesives [4].

In 2012, however, Hanabusa et al. [17] reported that the μTBS to dentine of G-bond Plus (GC Corporation) applied following “wet bonding” in the etch-and-rinse protocol was significantly lower than that when the G-bond Plus was applied following “dry-bonding” in the etch-and-rinse protocol. The authors mentioned that G-Bond Plus, which is a one-step self-etch adhesive, does not require a wet bonding technique because the adhesive contains water to enable the self-etching potential [17]. This is partly in agreement with the results of the present study as the dry bonding in etch-and-rinse mode did not result in a significantly lower μTBS for GPB and SBU. The water content of the adhesives is strongly related to their pH because it is essential to ionize the acidic functional monomers and make self-etching possible [20,21]. The more aggressive self-etch adhesives, which contain substantial amounts of highly acidic resin monomers, are likely to contain a larger amount of water [22]. The water content of the universal adhesives tested was approximately 25% for GPB (pH = 1.5) [23], 10% for SBU (pH = 2.7) [24], and less than 3% for ABU (pH = 3.2) [25]. In addition, when the dentin surfaces are desiccated, the amount of water present in the adhesives must be used both for the initial ionization of the acidic component and for wetting the desiccated dentin surfaces. In this regard, GPB and SBU were capable of re-expanding the air-dried and collapsed collagen mesh to facilitate adhesive resin infiltration, but ABU was unable to re-expand the excessively air-dried and collapsed collagen mesh because of its low water content. For the ABU in etch-and-rinse mode, CLSM showed that the resin tags were dispersed more individually at the resin/dentin interface, and the penetration depth was relatively shallow for the 10 s group compared with the 0 s and 5 s groups (Figure 5). Such poorly resin-infiltrated zones observed in the selected samples of ABU when the dentin surfaces were air-dried for more than 10 s might have affected the mechanical stability of the bond, which would explain the significantly reduced μTBS outcome recorded in this study.

In the self-etch strategy, the etching effect of the self-etch adhesives is related to the acidic functional monomers that interact with the mineral component of the tooth substrate and form a continuum between the tooth surface and adhesive by the simultaneous demineralization and resin infiltration. Consequently, the adhesives need to contain water and water-soluble hydrophilic monomers, such as 2-hydroxyethyl methacrylate (HEMA), so that the acidic monomer can dissociate and penetrate into the hydrophilic dentin [17]. Previous studies have examined the effects of the dentin wetness on the bond strength of simplified adhesive systems. Hashimoto et al. evaluated in vitro the effect of water on the dentin substrate bonding of one-bottle self-etching adhesives and found that the bond strength of the dry-dentin group was significantly greater than that of wet-dentin [26]. More recently, they concluded that the bond strength depended on the wetness of the bonding substrate for both adhesive groups, with a positive effect on the bond strength for one-bottle adhesives and a negative effect for two-bottle self-etching adhesives [27]. According to the authors, water movement in the resin/dentin interface occurs immediately after an air blast or light curing during the bonding procedures. The hydrophilic adhesives could be diluted by this rapid and large water movement because dentin is a wet substrate [28]. When the adhesive is applied on the dentin surface, fluid movement also occurs due to the osmotic pressure, and this osmotic water movement may occur convectively because the primers or adhesives have a relatively high osmorality [29]. Although water is an essential component in adhesives to generate the hydrogen ions needed for effective dissolution and demineralization [30], excessive water can lead to monomer dilution and/or inhibit resin polymerization [31]. Consequently, the water sorption from dentin might increase the elastic buffering effect of adhesives and result in a decrease of bond strength [27]. This could lead to a decrease in bond strength for ABU when the dentin surfaces are moist. In CLSM analysis, however, the morphology of the adhesive interfaces of the three universal adhesives was similar (Figure 5 and Figure 6). These results are consistent with previous studies that reported that the pH of self-etching adhesives does not influence the morphology of the dentin–resin interfaces [32,33]. In the present study, the morphology of adhesive interfaces was also similar in the experimental groups for each adhesive.

In addition to the pH of the adhesives, the solvent chemistry and specific functional monomer types and ratios could also play a vital role in these adhesives [34,35]. The surface wetness essential for proper dentin bonding varies according to the solvent type and amount of water present in each adhesive. Several studies have reported that acetone-based adhesive systems are more dependent on an accurate wet bonding technique than ethanol-based adhesives [36,37,38,39]. Nevertheless, the high bond strength of dentin can be obtained under wet and dry dentin conditions when acetone-based systems are agitated on the dentin surface [30]. Interestingly, the acetone-based GPB did not show a significant difference in the mean μTBS under different moist conditions after acid etching, even though GPB was not agitated when applied to the dentin surfaces according to the manufacturer’s instructions. One possible explanation for this result is that either the difference in the solvent concentrations or monomer composition in GPB could affect the results. On the other hand, SBU is an ethanol/water-based solvent adhesive, containing the optimal concentration of ethanol as a solvent for this adhesive formulation to allow a decrease in viscosity that facilitates wetting of the surface and penetration of the adhesive into wet and dry dentin substrates [31]. SBU also contains a vitrebond copolymer, which provides stability against humidity deterioration [40], and could be associated with the present results.

Considering the results of the present study, as dentin wetness significantly influences the μTBS of universal adhesive products dependently, clinicians should carefully control the moisture of dentin surfaces with special consideration for the product.

Nevertheless, these findings should be interpreted with caution because the water present during the bonding procedures could originate from not only the rinsing procedures and adhesive solutions, but also from the intrinsic dentinal fluid and atmospheric water (i.e., relative humidity) [41]. Due to the presence of residual solvent and fluid movement in dentinal tubules, infiltration of resin into water-filled collagen fibril matrices is incomplete [1]. The results in this study were obtained under laboratory conditions and the dentin perfusion that varies with the depth of dentin could not be simulated. Moreover, the degradation that occurs at the adhesive interface is also of critical importance for the longevity of the restorations [42]. On the other hand, it is well known that long term water exposure can promote bond degradation [43,44]. Water storage can lead to degradation of the interface components by hydrolysis of collagen and/or elution of insufficiently cured resin [45]. Water that infiltrates into the interface components can also cause a decrease in the mechanical properties of the polymer matrix by plasticization of the resin [46,47]. Although most universal adhesives contain 10-MDP or other organophosphate monomers that can produce chemical adhesion to the tooth substrates [17], the dilution of adhesives by water penetration and monomer diffusion at the wet-dentin/adhesive interface can lead to a low degree of conversion of the adhesive resin and a weak hybrid layer that is susceptible to degradation by aging [48]. Therefore, the long-term performance of the bonded interfaces needs to be evaluated further.

## 4. Materials and Methods

### 4.1. Tooth Selection and Preparation

Seventy-two extracted, caries-free human third molars within three months of extraction were used. This study was approved by the Institutional Review Board of Pusan National University Dental Hospital (IRB, PNUDH-2015-020). The teeth were disinfected with 0.5% chloramine T, and stored in distilled water at 4 °C until needed. The teeth were sectioned horizontally at the mid-coronal level to obtain flat, sound dentin surfaces with a water-cooled diamond saw (Accutom-50, Struers, Rødovre, Denmark). The exposed dentin surfaces were wet-polished further with 600-grit SiC paper for 60 s to standardize the smear layer.

### 4.2. Experimental Design and Specimen Preparation

The teeth were assigned randomly to six groups according to the different bonding strategies (etch-and-rinse or self-etch mode) of the selected universal adhesive systems: G-Premio Bond (GPB; GC Corp., Tokyo, Japan), Single Bond Universal (SBU; 3M ESPE, St. Paul, MN, USA), and All Bond Universal (ABU; Bisco Inc., Shaumburg, IL, USA). Each group was divided into three subgroups according to the air-drying time of the dentin surfaces: 0 (moist dentin surfaces without air drying), 5, and 10 s. The moist dentin surface was obtained by absorbing excessive water using a cotton pellet. In contrast to the dentin surfaces of the etch-and-rinse mode where the dentin surfaces were dried after etching with 37% phosphoric acid, the dentin surfaces of the self-etch mode were dried before applying the adhesives because the self-etch mode does not need a separate acid etching step. The dentin surface was dried using a three-way air syringe with the air pressure adjusted to 1 bar using a pressure regulator, and the air nozzle was held at 45° to the dentin surface at a distance of 1.5 cm. The adhesives were applied in etch-and-rinse and self-etch mode according to the manufacturer's instructions, as listed in Table 2. After the bonding procedures, composite resin (Filtek Z250, 3M ESPE, St. Paul, MN, USA) was placed on the dentin surface in two increments of 2 mm each. Each increment was light-cured for 20 s using a light-emitting diode (LED) dental light-curing unit set to 1200 mW/cm^2^ (BluephaseG2, Ivoclar Vivadent Inc., Amherst, NY, USA). A radiometer (Demetron LED Radiometer, Kerr Sybron Dental Specialties, Middleton, WI, USA) was used to examine the light intensity after completing five specimens.

### 4.3. Microtensile Bond Strength (μTBS)

After storage of the bonded teeth in distilled water at 37 °C for 24 h, the teeth were sectioned longitudinally in both the “x” and “y” directions across the bonded interface with a water-cooled diamond saw in a cutting machine (Accutom-50, Struers, Rødovre, Denmark) to produce sticks with a cross-sectional area of approximately 1 mm^2^. The sticks were examined carefully with a stereomicroscope at 30× magnification and those with defects at the resin/dentin interface were discarded. The resin–dentin bonded sticks were attached to a jig with cyanoacrylate cement (Zapit, Dental Ventures of America, Corona, CA, USA) and subjected to a microtensile test in a universal testing machine (Bisco, Schaumburg, IL, USA) at a crosshead speed of 0.5 mm/min until failure. The μTBS values were calculated by dividing the load at failure by the cross-sectional bonding area.

### 4.4. Failure Mode Analysis

After the test, all debonded sticks were observed under a stereomicroscope at ×40 magnification (Leica, Heidelberg, Germany) to determine the failure mode. Premature failure (PF) was considered to be a pre-testing failure caused by the specimen preparation. The failure mode was classified as cohesive (C, exclusively within dentin or resin composite), adhesive (A, at the resin/dentin interface), or mixed (M, at the resin/dentin interface that included cohesive failure of the neighboring substrates). Specimens with premature failure for cohesive failure were excluded.

### 4.5. Confocal Laser Scanning Microscopy Analysis

Rhodamine B fluorescent dye (Merck, Darmstadt, Germany) at a concentration of 0.01 wt % was added to the bonding agent prior to application to the dentin surfaces. Using the same adhesive protocols described above, one specimen per group was prepared for observation under confocal laser scanning microscopy (CLSM; TCS SL, Leica, Wetzlar, Germany). Only one composite increment was placed on the adhesive resin. The teeth were then cut longitudinally into two halves and both surfaces were wet-polished for one minute with SiC paper in sequence (Grit 800/1200/2000/4000). The samples were washed thoroughly with water and kept moist prior to the CLSM examination. A CLS microscope was used to obtain images of the bonded interfaces, focusing on the thickness of the bonding agent, formation of a hybrid layer, and penetration of the bonding solution into the dentinal tubules. The fluorescent images were obtained with LSM-700 (Carl Zeiss, Oberkochen, Germany) in a 200-fold magnification and processed with Image J (NIH, Bethesda, MD, USA).

### 4.6. Statistical Analysis

Specimens with premature failure were not included in data analysis of the μTBS because of their low frequency in the experiment. The μTBS data were analyzed using a two-way ANOVA followed by a Tukey’s *post hoc* test at a 5% level of significance (SPSS 21.0; SPSS Inc., Chicago, IL, USA). A one-way ANOVA and a Scheffe’s multiple comparison test (α < 0.05) were also used to compare each condition individually, regardless of the adhesive or moisture condition. The adhesive interfaces were evaluated only qualitatively.

## 5. Conclusions

Within the limitation of this in vitro study, the wetness of the dentin surface is an influencing factor that can affect the dentin bond strength depending on the universal adhesive system used.

## Figures and Tables

**Table 1:** Microtensile bond strength values (MPa) means and standard deviation for all experimental groups.

**Adhesive**	**Etch-and-Rinse Mode**			
	**0 s**	**5 s**	**10 s**	***p*-value**
GPB	33.2 ± 3.3 ^a^	35.2 ± 4.5 ^a^	34.1 ± 2.4 ^a^	0.868
SBU	31.8 ± 3.9 ^a^	35.4 ± 4.9 ^a^	32.1 ± 4.0 ^a^	0.105
ABU	32.0 ± 3.1 ^a^	31.8 ± 2.6 ^a^	23.4 ± 2.2 ^b^	<0.001
**Adhesive**	**Self-Etch Mode**			
	**0 s**	**5 s**	**10 s**	***p*-value**
GPB	32.8 ± 4.3 ^a^	32.4 ± 2.6 ^a^	33.4 ± 1.9 ^a^	0.760
SBU	29.0 ± 3.6 ^a^	31.1 ± 4.9 ^a^	28.2 ± 2.8 ^a^	0.226
ABU	17.4 ± 2.6 ^a^	22.7 ± 5.5 ^b^	22.8 ± 2.5 ^b^	0.003

GBU: G-Premio bond; SBU: Single bond universal; ABU: All-bond universal. Different superscript lowercase letters indicate significant differences between rows (*p* < 0.05); i.e., excluding the premature failure samples and those broken within dentine or with glue covering the binding site.

**Table 2:** Adhesive composition and application procedure (information supplied in the safety data sheets and material instructions).

Material	pH	Composition	Application Mode	
			Self-Etch	Etch-and-Rinse
G-Premio Bond (GPB) GC Corp. Tokyo, Japan P	1.5	10-MDP, phosphoric acid ester monomer, dimethacrylate, 4-MET, MEPS, acetone, silicon dioxide, initiators	1. The only difference is the dentin drying time: 0, 5, and 10 s 2. Apply the adhesive to the entire preparation without rubbing for 10 s 3. Direct a gentle stream of air over the liquid for about 5 s until it no longer moves and the solvent is evaporated completely	1. Apply etchant for 15 s 2. Rinse thoroughly for 5 s 3. The only difference is the dentin drying time: 0, 5, and 10 s 4. Apply adhesive as in the self-etch mode
Single-bond Universal (SBU) 3M ESPE Seefeld, Germany	2.7	10-MDP, phosphoric acid ester monomer, HEMA, silane, dimethacrylate, Vitrebond copolymer, filler, ethanol, water, initiators, silane	1. The only difference is the dentin drying time: 0, 5, and 10 s 2. Apply the adhesive to the entire preparation with a microbrush and rub it in for 20 s 3. Direct a gentle stream of air over the liquid for about 5 s until it no longer moves and the solvent is evaporated completely 4. Light-cure for 10 s	1. Apply etchant for 15 s 2. Rinse thoroughly for 10 s 3. The only difference is the dentin drying time: 0, 5, and 10 s 4. Apply adhesive as in the self-etch mode
All-Bond Universal (ABU) Bisco Schaumburg, USA	3.2	10-MDP, phosphoric acid ester monomer, Bis-GMA, HEMA, ethanol, water, initiators	1. The only difference is the dentin drying time: 0, 5, and 10 s 2. Apply two separate coats of adhesive, scrubbing the preparation with a microbrush for 10–15 s per coat. Do not light cure between coats 3. Evaporate excess solvent by thoroughly air-drying with an air syringe for at least 10 s, there should be no visible movement of the material. The surface should have a uniform glossy appearance 4. Light cure for 10 s	1. Apply etchant for 15 s 2. Rinse thoroughly for 10 s 3. The only difference is the dentin drying time: 0, 5, and 10 s 4. Apply adhesive as in the self-etch mode

Bis-GMA: bisphenol glycidyl methacrylate; HEMA: 2-hydroxyethyl methacrylate; MDP: methacryloyloxydecyl dihydrogen phosphate; 4-MET: 4 methacryloxyethyltrimellitate anhydride; MEPS: Methacryloyloxyalkyl thiophosphate.
